# Promoting physical activity in a multi-ethnic population at high risk of diabetes: the 48-month PROPELS randomised controlled trial

**DOI:** 10.1186/s12916-021-01997-4

**Published:** 2021-06-03

**Authors:** Kamlesh Khunti, Simon Griffin, Alan Brennan, Helen Dallosso, Melanie J. Davies, Helen C. Eborall, Charlotte L. Edwardson, Laura J. Gray, Wendy Hardeman, Laura Heathcote, Joe Henson, Daniel Pollard, Stephen J. Sharp, Stephen Sutton, Jacqui Troughton, Tom Yates

**Affiliations:** 1 Diabetes Research Centre, College of Medicine, Biological Sciences and Psychology, University of Leicester, Leicester, UK; 2 NIHR Applied Research Collaboration - East Midlands, Leicester, UK; 3 MRC Epidemiology Unit, Institute of Metabolic Science, University of Cambridge, Cambridge, UK; 4 Primary Care Unit, Department of Public Health and Primary Care, University of Cambridge, Cambridge, UK; 5 School of Health and Related Research, University of Sheffield, Sheffield, UK; 6 Leicester Diabetes Centre, University Hospitals of Leicester NHS Trust, Leicester, UK; 7 NIHR Leicester Biomedical Research Centre, University Hospitals of Leicester NHS Trust and University of Leicester, Leicester, UK; 8 Usher Institute, University of Edinburgh, Edinburgh, UK; 9 Biostatistics Research Group, Department of Health Sciences, University of Leicester, Leicester, UK; 10 School of Health Sciences, University of East Anglia, Norwich, UK; 11 Behavioural Science Group, Primary Care Unit, Department of Public Health and Primary Care, University of Cambridge, Cambridge, UK

**Keywords:** Diabetes prevention, mHealth, Randomised controlled trial, Non-diabetic hyperglycaemia, Group-based intervention, Physical activity, Pedometer

## Abstract

**Background:**

Physical activity is associated with a reduced risk of type 2 diabetes and cardiovascular disease but limited evidence exists for the sustained promotion of increased physical activity within diabetes prevention trials. The aim of the study was to investigate the long-term effectiveness of the Walking Away programme, an established group-based behavioural physical activity intervention with pedometer use, when delivered alone or with a supporting mHealth intervention.

**Methods:**

Those at risk of diabetes (nondiabetic hyperglycaemia) were recruited from primary care, 2013–2015, and randomised to (1) Control (information leaflet); (2) Walking Away (WA), a structured group education session followed by annual group-based support; or (3) Walking Away Plus (WAP), comprising WA annual group-based support and an mHealth intervention delivering tailored text messages supported by telephone calls. Follow-up was conducted at 12 and 48 months. The primary outcome was accelerometer measured ambulatory activity (steps/day). Change in primary outcome was analysed using analysis of covariance with adjustment for baseline, randomisation and stratification variables.

**Results:**

One thousand three hundred sixty-six individuals were randomised (median age = 61 years, ambulatory activity = 6638 steps/day, women = 49%, ethnic minorities = 28%). Accelerometer data were available for 1017 (74%) individuals at 12 months and 993 (73%) at 48 months. At 12 months, WAP increased their ambulatory activity by 547 (97.5% CI 211, 882) steps/day compared to control and were 1.61 (97.5% CI 1.05, 2.45) times more likely to achieve 150 min/week of moderate-to-vigorous physical activity. Differences were not maintained at 48 months. WA was no different to control at 12 or 48 months. Secondary anthropometric and health outcomes were largely unaltered in both intervention groups apart from small reductions in body weight in WA (~ 1 kg) at 12- and 48-month follow-up.

**Conclusions:**

Combining a pragmatic group-based intervention with text messaging and telephone support resulted in modest changes to physical activity at 12 months, but changes were not maintained at 48 months.

**Trial registration:**

ISRCTN 83465245 (registered on 14 June 2012).

**Supplementary Information:**

The online version contains supplementary material available at 10.1186/s12916-021-01997-4.

## Background

The rising burden of type 2 diabetes (T2D) has precipitated three decades of research and healthcare policies concerning prevention among individuals deemed to be at risk. Large trials have demonstrated that intensive lifestyle interventions targeting diet, physical activity and weight loss reduce the risk of developing T2D by 50% [1]. Translational research has demonstrated that lifestyle diabetes prevention programmes also lead to modest weight loss when implemented within routine clinical settings [2]. This has led to commissioning and delivery of lifestyle advice and diabetes prevention programmes within routine health care settings [3, 4].

Whilst the intensive interventions in the seminal diabetes prevention trials achieved initial weight loss, there is little evidence of sustained increases in physical activity over the longer term (> 12 months) [5]. This is important as even modest increases in physical activity decrease the risk of cardiovascular disease and improve glycaemic control independently of changes in weight in high risk groups [6, 7] and facilitate maintenance of weight loss. Furthermore, uptake of and retention in real-world diabetes prevention programmes is sub-optimal [3, 4], suggesting alternative strategies are required.

The Walking Away from type 2 diabetes programme (referred to hereinafter as “Walking Away”) is a 3-h group-based structured education programme with annual refresher sessions that was developed for implementation within family practice and has been widely commissioned into routine care [8]. An early trial demonstrated small changes in physical activity over 12 months, but with evidence of greater behaviour change in those with nondiabetic hyperglycaemia [8].

The PROPELS trial investigated the longer-term effectiveness of Walking Away in a multi-ethnic population with nondiabetic hyperglycaemia, when delivered in a standard format or when integrated with a bespoke mHealth intervention designed to maintain physical activity behaviour change.

## Methods

The PRomotion Of Physical activity through structured Education with differing Levels of ongoing Support for those with prediabetes (PROPELS) study is a multi-centre, open, individually randomised three-arm trial, described in the published protocol [9]. Ethical approval was granted by the NHS National Research Ethics Service, East-Midlands Leicester Committee (Ethics number: 12/EM/0151). Participant recruitment commenced in December 2013 and was completed in February 2015, with follow-up data collection completed in July 2019.

### Recruitment of participants

Participants were recruited from the East Midlands and Eastern regions of England, purposefully targeting areas with large multi-ethnic communities. The primary method of recruitment was through family practice, supplemented by recruitment from research databases.

Age eligibility was 40 to 74 years for White Europeans, or 25–74 years for those from an ethnic minority to account for higher diabetes risk status and to comply with national guidelines [10]. Additional eligibility criteria were previously recorded plasma glucose or HbA_1c_ value in the nondiabetic hyperglycaemia range (HbA_1c_ ≥ 42 [6.0], < 48 [6.5] mmol/mol [%]; fasting glucose ≥ 5.5, < 7.0 mmol/l; 2-h post-challenge glucose ≥ 7.8, < 11.1 mmol/l) within the last 5 years, and access to a mobile phone. Individuals unable to take part in ambulatory-based activity, were pregnant, diagnosed with diabetes or non-English speakers were excluded.

### Randomisation and blinding

Participants were randomised (stratified by centre [Leicester vs. Cambridge], sex and ethnicity [White European vs. other]) using an online randomisation tool (https://www.sealedenvelope.com/) through the University of Leicester Clinical Trials Unit. Individuals were randomised (1:1:1) to one of three groups: Control, Walking Away (WA) or Walking Away Plus (WAP). Allocation was not blinded due to the nature of the trial. However, study allocation was concealed from the study measurement and laboratory teams and the research staff processing the accelerometer data (primary outcome).

#### Control

Participants allocated to control received an advice leaflet targeting knowledge of nondiabetic hyperglycaemia and highlighting the importance of physical activity.

#### Walking Away (WA)

WA is a 3-h group-based, theory-driven, behavioural intervention addressing knowledge and perceptions of diabetes risk and promoting increased physical activity; the theoretical underpinning, content and structure of the intervention has been described previously [9]. The central aim is to promote increases of physical activity up to 3000 steps/day. Goal attainment is encouraged through the provision of pedometers (Yamax SW200) and step/day dairies. A short section of the curriculum is also allocated to covering key dietary messages.

WA sessions were delivered by two trained educators following a structured curriculum to groups of up 10 participants. Sessions were delivered in a variety of settings chosen for proximity to recruiting family practices, including the practices themselves, in nearby community centres or at hospital sites.

Participants were offered annual group-based follow-on maintenance sessions at 12, 24 and 36 months. Annual follow-on sessions lasted 2.5 h and were designed to revisit the key messages of the initial session, strengthen self-efficacy through sharing successes and prompt problem-solving in relation to barriers, goal setting and pedometer use.

#### Walking Away Plus (WAP)

Participants assigned to WAP were invited to attend the same WA session and annual refresher sessions as described above [9, 11]. In addition, they received an mHealth follow-on support intervention which was based on prompting participants by text to set goals and to text back step counts. Automated feedback was then texted to participants with the content tailored to success with achieving goals and other individual tailoring characteristics such as self-efficacy that were captured during an initial telephone call with trained staff within a week of attending WA. The content of the automated text messages were developed for use with Walking Away, as described previously [9, 11]. Text messages were sent at least weekly over the first 6 months and then monthly. Participants could opt out of receiving texts. Participants also received a further telephone call at six months to review progress. The telephone call and text message frequency was repeated after each annual group-based follow-on session [9].

### Primary outcome measure

The primary outcome was change in ambulatory activity (steps/day) at 48 months, assessed by accelerometer (Actigraph GT3X+), with an intermediary assessment at 12 months. Participants were asked to wear the accelerometer on a waistband (on the right anterior axillary line) during waking hours for seven consecutive days.

Acceleration data were integrated into 60-s epochs. At least 3 days valid wear (≥ 10 h of data per day) were required for inclusion in the analysis. Non-wear time was determined by one hour or more of consecutive zero counts.

Actigraph accelerometers have previously been shown to produce valid measures of steps taken during treadmill and free-living walking [12, 13], particularly for moderate and brisk stepping where intraclass correlation coefficients compared to criterion measures have been shown to be > 0.9.

### Secondary outcomes

The accelerometer used to measure the primary outcome also measured censored ambulatory activity, defined as steps taken above an intensity (500 counts/minute) distinguishing between purposeful and incidental ambulation [14]. Freedson cut-points distinguished between time spent sedentary, in light-intensity physical activity and in moderate-to-vigorous intensity physical activity (MVPA) [15]. Compliance with physical activity recommendations (undertaking at least 150 min of MVPA per week) was also assessed as total MVPA or that undertaken in at least 10-min bouts.

Participants were also asked to wear an activPAL3™ device, attached to the thigh to determine time spent sitting, standing and stepping. Data were analysed using an open-source processing package (ProcessingPAL, University of Leicester https://github.com/UOL-COLS/ProcessingPAL).

Self-reported physical activity energy expenditure was measured using the validated recent physical activity questionnaire [16]. Sleep duration was assessed by self-report (last night and average duration) [9]. HbA_1c_, lipid profile (triglycerides, HDL, LDL, total cholesterol), urea and electrolytes (sodium, potassium, urea, creatinine) and liver function tests (albumin, total bilirubin, alkaline phosphatase, alanine transaminase) were assessed using venous samples. During the course of the trial, those found to have diabetes (HbA_1c_ ≥ 6.5% or 48 mmol/mol) continued to be offered all study and interventional procedures.

Information on ethnicity was obtained by self-report. We calculated modelled cardiovascular risk using the Framingham Risk Score. Social deprivation was assessed using the Index of Multiple Deprivation (IMD) score derived for each participant’s postcode.

Dietary behaviour was measured by an abbreviated food frequency questionnaire developed for the European Prospective Investigation of Cancer and Nutrition (EPIC) study and a questionnaire of dietary intentions developed for the NAVIGATOR (Nateglinide And Valsartan in Impaired Glucose Tolerance Outcomes Research) study [17, 18].

We measured health-related quality of life using the European Quality of Life-5 Dimensions (EQ-5D-5L) and the Short Form (SF-8). Depression and anxiety were assessed using the Hospital Anxiety and Depression Scale (HADS) [9], medical history and medication status by interview administered protocol and family history of diabetes and cardiovascular disease, smoking status and muscular/skeletal injury were assessed by self-report. All adverse events reported to the study sponsor (University of Leicester) were recorded.

### Family practice data

We collected data on biochemistry, diabetes diagnosis and other medical events that occurred during the trial directly from consenting participants’ family practice records for those lost to follow-up.

### Mediators of behaviour change

The Brief Illness Perceptions Questionnaire (BIPQ) was used to measure perceptions and perceived knowledge of diabetes risk [9]. Participants’ confidence in their ability to walk for 10, 30 and 60 min each day was assessed using rating scales (ranging from 0% [no confidence] to 100 % [complete confidence]) [9]. The use of behaviour change strategies at 12 and 48 months were assessed using a 5-point Likert scale. Items assessed included how often participants set goals, formed action plans, used a pedometer, completed a physical activity log, were aware of their activity levels and were trying to be more physically active [9].

### Sample size

Assuming a 2.5% significance level (allowing for two a priori comparisons of WA and WAP against control) and 80% power, based on an SD of 4000 steps/day over 4 years [9], 918 participants (306 per group) were required to complete the trial in order to detect a 1000 steps/day difference in change in ambulatory activity. Allowing for 30% loss to follow-up or incomplete primary outcome data, the recruitment target was 1308.

### Statistical analysis

The statistical analysis plan was published on the trial registry (ISRCTN 83465245) before unblinding of data. We compared change in the primary outcome between each intervention group and the control group using analysis of covariance (ANCOVA) with adjustment for baseline, randomisation stratification variables (centre, ethnicity, sex). Accelerometer outcomes were also adjusted for wear time at baseline and follow-up, and number of valid days of wear at baseline and follow-up. Data on illness perception, self-efficacy and self-reported use of behaviour change strategies were summarised descriptively.

In order to investigate the potential impact of missing data, further analyses of the primary outcome were performed using multiple imputation by chained equations (also assuming MAR), and a pattern mixture model, to investigate departures from the MAR assumption [19].

For the primary outcome, pre-specified interactions between randomised group and the following baseline variables were investigated: ethnicity (White European/South Asian/Other), sex (men/women), age (< 60 years/≥ 60 years), family history of T2D (yes/no), nondiabetic hyperglycaemia (yes/no), obesity status (< 30 kg/m^2^ [27.5 kg/m^2^ for South Asians], ≥ 30 kg/m^2^ [27.5 kg/m^2^ for South Asians]) and deprivation (split at median IMD score into high vs low).

A per-protocol analysis was conducted according to the following criteria:

Control – all individuals.

WA – attended initial session AND at least 1 follow-up annual refresher session.

WAP – attended initial session AND at least 1 follow-up annual refresher session AND registered with the text service AND received the initial telephone call AND received at least one further telephone call during the trial.

Significance was set at *p* < 0.025 for main effects with results reported as mean (97.5% CI) to account for multiple testing and *P* < 0.05 for interactions. Analyses were performed using Stata version 15.1 (StataCorp 2017)

## Results

**Fig. 1:**
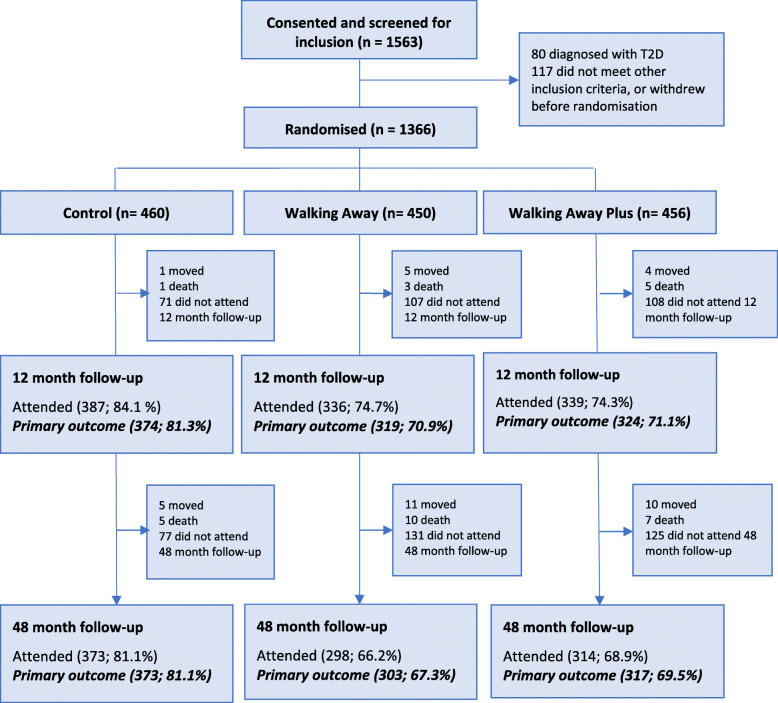
Participant flow

Invitation letters were sent to 12,417 individuals from 47 different family practices, with a further 746 invited from previous research databases. Of these, 1563 individuals provided consent and were screened, with 1366 meeting the inclusion criteria and randomised. The flow of participants is shown in Fig. 1. The sociodemographic and clinical characteristics of participants, stratified by randomised group, are presented in Table 1; 28% were from black and minority ethnic populations. Primary outcome data at 48-month follow-up were available for 993 (72.7%). The characteristics of those with and without primary outcome data, stratified by intervention group, are shown in Additional File 1.

**Table 1:** Sociodemographic and clinical characteristics of participants, stratified by randomised group

Participant characteristics	Control (*N*=460)		Walking Away (*N*=450)		Walking Away Plus (*N*=456)	
Continuous variables	Mean	SD	Mean	SD	Mean	SD
Age (yrs)	59.4	8.8	59.4	9.4	59.3	9.1
BMI (kg/m^2^)	28.5	5.7	28.2	5.6	28.4	5.6
Social deprivation (IMD decile)	5.5	2.8	5.7	3.0	5.7	2.8
HbA1c (mmol/mol)	5.8	0.3	5.9	0.4	5.9	0.3
HbA1c (%)	40.0	3.7	40.5	3.5	40.4	3.5
Categorical variables	**%**	***n***	**%**	***n***	**%**	***n***
Sex						
Men	50.9	234	50.4	227	50.9	232
Women	49.1	226	49.6	223	49.1	224
Ethnicity						
White European	71.1	327	72.4	326	72.1	329
South Asian	22.4	103	22.0	99	22.6	103
Other	6.5	30	5.6	25	5.3	24
Family history of diabetes in first-degree relatives	43.3	198	42.0	188	45.3	205
Antihypertensive medication	40.9	169	44.6	164	44.7	170
Lipid-lowering medication	34.9	144	37.2	137	39.6	150
Steroids	7.4	34	9.1	41	6.4	29
Metformin	0.0	0	0.2	1	0.2	1
CVD (MI, heart failure, angina, stroke)	8.6	39	9.0	40	9.9	45
Smoking status						
Past	38.3	176	36.2	163	38.2	174
Current	9.8	45	8.4	38	11.4	52
Employment type						
Full time	37.6	173	34.2	154	37.1	169
Part time	16.1	74	20.4	92	18.9	86
Retired	35.0	161	35.3	159	33.6	153
Unemployed or other	11.3	52	10.0	45	10.5	48
Educational status						
Degree, higher degree or equivalent	45.7	205	45.5	197	44.9	202
Marital status						
Married/civil partner	68.3	314	75.6	340	73.9	337
Access to the internet	83.0	380	86.2	387	85.3	388
Meeting physical activity recommendations	53.7	238	56.1	245	57.3	254
Meeting physical activity recommendations in 10-min bouts	21.9	97	25.9	113	24.6	109

### Intervention engagement and adherence

Intervention engagement for each intervention group is shown in Table 2. Approximately 80% attended the initial WA session in both groups, and over two thirds attended at least one annual group-based follow-on session. There was also reasonable engagement with the key elements of the mHealth intervention in WAP (Additional file 2). At 48 months, 64.2% in WAP and 49.7% in WA still reported using their pedometer at least some of the time. Similarly, 40.9% and 30.6% in WAP and WA respectively reported keeping a physical activity log at least some of the time, compared to 11.1% in the control group. Self-efficacy for walking was high at baseline in all groups and remained high throughout the trial (Additional file 3). Illness perception scores indicated WA and WAP increased perceived understanding of diabetes risk over the course of the trial, whereas understanding remained stable in the control group (Additional file 3).

**Table 2:** Engagement with key components of the intervention

	Walking Away (*N*=450)		Walking Away Plus (*N*=456)	
Programme attendance	%	*n*	%	*n*
Attended initial education session	79.3	357	80.9	369
Attended 12-month refresher session	57.3	258	60.3	275
Attended 24-month refresher session	49.6	223	55.5	253
Attended 36-month refresher session	48.9	220	50.4	230
Attended at least 1 follow-up annual support session	67.6	304	69.7	318
Phone call and text messaging intervention				
Registered with text service			77.6	354
Received initial telephone call			69.1	315
Received at least 1 telephone call during the trial			85.1	388
Asked for text messaging service to be stopped			18.9	67

### Primary outcome

**Fig. 2:**
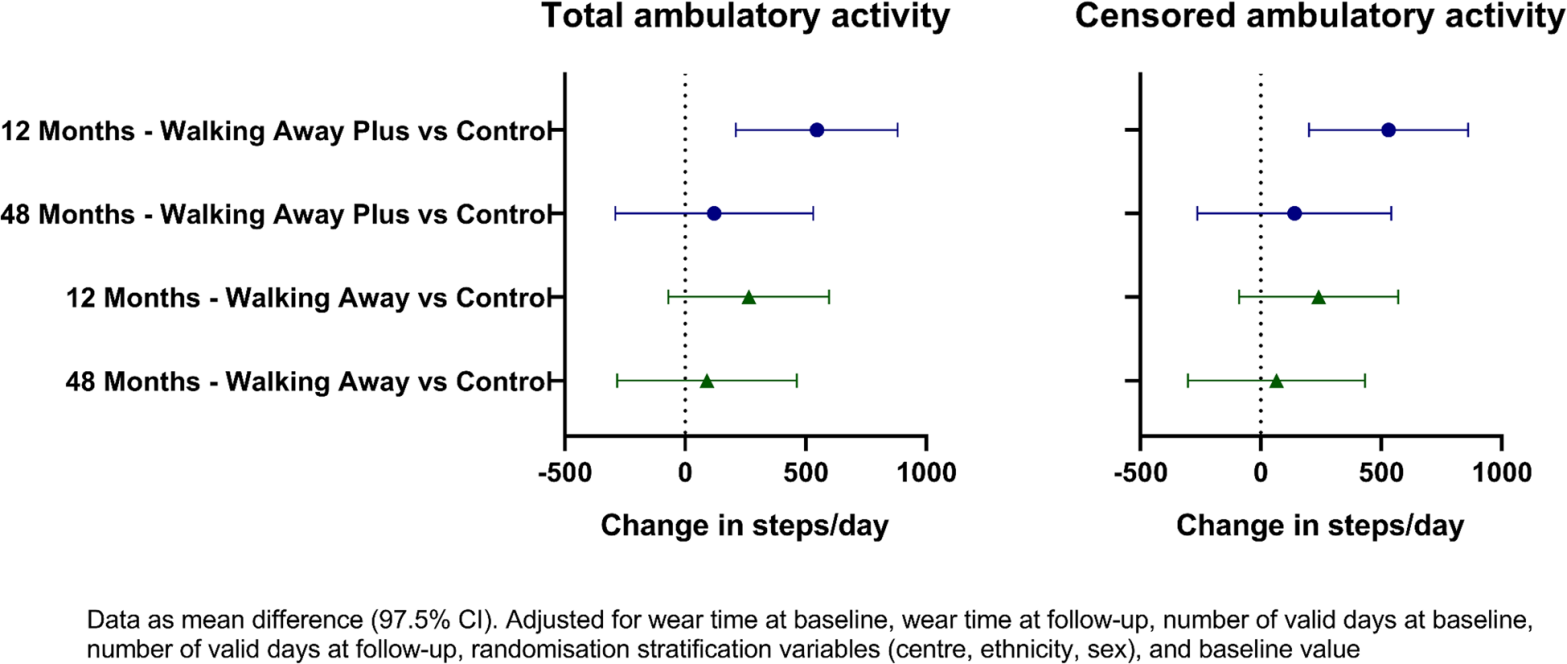
Change in ambulatory activity in intervention groups compared to control at follow-up

Total ambulatory activity (primary outcome) and physical variables at baseline and subsequent 12- and 48-month change values are presented in Table 3 and Fig. 2. At baseline, the control, WA and WAP groups took an average (SD) of 6885 (3068), 7264 (3009) and 7353 (3432) steps/day, respectively. WAP increased total ambulatory activity at 12 months by 547 (97.5% CI 211, 882) steps/day relative to control (Fig. 2). The results for total ambulatory activity were consistent with those for censored ambulatory activity (Fig. 2), indicating the increase was due to purposeful movement. No change in either group was found at 48 months compared to control (WA vs control 91 [− 282, 463] steps/day, WAP vs control 121 [− 290, 532] steps/day).

**Table 3:** Baseline and change values for objectively assessed physical activity and sedentary behaviour outcomes

	Control			Walking Away			Walking Away Plus			Intervention effect 1^c^ (Walking Away vs Control)			Intervention effect 2^c^ (Walking Away vs Control)		
	*N*	Mean	SD	*N*	Mean	SD	*N*	Mean	SD	Difference	97.5% CI		Difference	97.5% CI	
											Lower	Upper		Lower	Upper
Primary outcome															
Total ambulatory activity (steps/day)^a^															
Baseline value	441	6885	3068	427	7264	3009	435	7353	3432						
Change at 12 months	374	− 192	1680	319	− 2	2386	324	241	2270	264	− 70	597	**547**	**211**	**882**
Change at 48 months	373	− 385	2217	303	− 312.5	2499	317	− 296	2969	91	− 282	463	121	− 290	532
Secondary outcomes															
Censored ambulatory activity (steps/day)^a^															
Baseline value	441	5369.5	2984.0	427	5643	2892	435	5765	3300						
Change at 12 months	374	− 192	1633	319	− 7.0	2369	324	228	2247	240	− 90	570	**531**	**201**	**861**
Change at 48 months	373	− 337	2157	303	− 285	2469	317	− 235	2916	66	− 302	433	140	− 263	542
Time spent sedentary (min/day)^a^															
Baseline value	441	557.0	92.9	427	544.0	91.3	435	544.5	97.2						
Change at 12 months	374	− 1.5	74.7	319	3.4	73.8	324	2.5	76.6	− 1.9	− 11.1	7.2	− 7.7	− 16.9	1.5
Change at 48 months	373	− 1.4	83.9	303	13.1	81.7	317	23.9	90.3	0.1	− 10.2	10.4	4.7	− 5.7	15.1
Time spent in light physical activity (mins/day)^a^															
Baseline value	441	293.3	80.7	427	310.9	85.7	435	309.0	88.9						
Change at 12 months	374	− 7.3	52.3	319	− 10.8	58.8	324	− 7.0	58.8	0.9	− 7.4	9.3	4.4	− 4.1	13.0
Change at 48 months	373	− 14.5	64.5	303	− 15.4	67.7	317	− 21.0	65.2	− 0.1	− 9.8	9.6	− 5.7	− 15.3	4.0
Time spent in moderate-to-vigorous physical activity (min/day)^a^															
Baseline value	441	29.8	24.7	427	31.4	25.7	435	32.1	27.6						
Change at 12 months	374	− 1.2	15.2	319	− 0.2	22.2	324	1.7	20.2	1.3	− 1.7	4.3	**3.5**	**0.6**	**6.5**
Change at 48 months	373	− 2.4	19.2	303	− 2.3	23.8	317	− 1.0	24.7	0.5	− 2.8	3.7	1.6	− 1.9	5.0
Time spent sitting or lying down (min/day)^b^															
Baseline value	337	549.3	111.6	333	535.7	113.3	323	545.6	115.3						
Change at 12 months	314	− 4.8	84.2	279	10.2	84.2	289	1.6	79.3	4.3	− 10.2	18.9	− 8.4	− 22.9	6.0
Change at 48 months	260	10.2	98.1	213	− 3.7	99.7	211	10.3	94.7	− 15.0	− 33.8	3.8	− 10.6	− 29.9	8.7
Time spent standing (min/day)^b^															
Baseline value	337	288.3	95.1	333	306.7	95.4	323	294.5	100.7						
Change at 12 months	314	− 2.8	61.6	279	− 11.1	66.3	289	− 6.8	57.7	− 6.2	− 18.5	6.1	0.3	− 11.8	12.5
Change at 48 months	260	− 12.8	77.5	213	0.8	78.5	211	− 7.4	73.6	12.7	− 2.7	28.1	5.9	− 9.9	21.6
Time spent stepping (mins/day) ^b^															
Baseline value	337	106.0	38.1	333	115.3	38.5	323	111.5	43.4						
Change at 12 months	314	− 1.4	22.8	279	0.9	29.3	289	4.8	27.3	2.4	− 2.8	7.6	**8.5**	**3.3**	**13.7**
Change at 48 months	260	− 3.7	28.9	213	− 3.2	28.7	211	− 3.7	37.1	2.2	− 4.3	8.6	4.8	− 2.5	12.0

^a^Data derived from waist worn accelerometer

^b^Data derived from thigh worn accelerometer

^c^Data adjusted for wear time at baseline, waking wear time at follow-up, number of valid days at baseline, number of valid days at follow-up, randomisation stratification variables (centre, ethnicity, sex), and baseline value. Bold indicates significance at *p* < 0.025

At 48 months, 278 (62%) in WA and 235 (52%) in WAP met the per-protocol definition; results were similar when analyses were restricted to this population (Additional file 4). Results for the primary outcome were also comparable following multiple imputation (Additional file 4), with the pattern mixture model showing similar conclusions even when there were substantial deviations from the MAR assumption. Furthermore, the results were consistent across sex, age, ethnicity, family history of diabetes and baseline prediabetes and obesity status (Additional file 5). However, there was evidence that the primary outcome was modified by social deprivation (*p* = 0.035 for interaction); in WAP compared to the control group, those below the median level of social deprivation had a decrease in activity level at 48 months (− 370 (− 945, 205) steps/day), whilst those above the median increased their ambulatory activity (480 (− 73, 1033) steps/day) (Additional file 5).

### Physical activity and sedentary behaviour

Time in MVPA increased by 3.5 (0.6, 6.5) min/day and time spent walking increased by 8.5 (3.3, 13.7) min/day in WAP compared to control at 12 months, but differences were not sustained at 48 months (Table 3). There were no differences between either intervention group compared to control in time spent in measures of sedentary behaviour, standing or in light-intensity physical activity (Table 3).

The odds of meeting the physical activity guidelines at 12 months was 1.61 [1.05, 2.45] times higher in WAP compared to control with similar results when considering time accumulated in at least 10-min bouts (OR = 1.63; 1.04, 2.55). However, no differences were observed at 48 months.

There was an increase in total self-reported physical activity energy expenditure in WAP compared to the control of 4.4 (0.0, 8.8) kJ/kg/day at 48 months (Additional file 6).

### Other secondary outcomes

Baseline values and the intervention effect at 12 and 48 months for all secondary outcomes are reported in Additional file 6. At 48 months in WA, there was a 1.00 (0.07, 1.92)kg reduction in body mass, a 1.57 (0.45, 2.70)cm reduction in waist circumference and a 1.06 (0.33, 1.79)% reduction in body fat percentage compared to control, with changes also observed at 12 months. Apart from a small decrease in triglycerides (− 0.15 mmol/l; − 0.29, − 0.01) in WAP at 48 months and a reduction in liver enzymes alanine aminotransferase (ALT) and alkaline phosphatase (ALP) in WA, there was no other clear pattern of differences between groups in clinical outcomes, depression or quality of life.

Both intervention groups reported increases in fresh fruit and vegetable consumption over the course of the trial; however, differences were small with increases of less than one portion a week compared to control (Additional file 6).

During the trial, 39 (9.3%) individuals in control, 30 (7.8%) individuals in WA and 41 (10.4%) individuals in WAP developed T2D with no difference in either intervention group compared to control.

The number of serious and non-serious adverse events in the control group was 7 (1.5%) and 47 (3.4%), respectively. Equivalent values for WA were 15 (3.3%) and 14 (3.11%), respectively, and for WAP 28 (6.4%) and 16 (3.5%), respectively. A breakdown of adverse event reporting in each group is displayed in Additional file 7.

## Discussion

Among people with previous nondiabetic hyperglycaemia, a pragmatic, 3-h group-based behavioural intervention, when combined with tailored text messages and telephone calls, increased ambulatory activity by over 500 steps/day or 8.5 min/day of walking after the first 12 months; however, effects were not maintained after 48 months. Results were similar in White European and Black and minority ethnic populations, although there was evidence that the most socially deprived were least likely to benefit.

The increase in ambulatory activity seen in the WAP group relative to control at 12 months, although modest, is likely to be clinically meaningful [20–22]. Although evidence from physical activity trials over 12 months is limited, the finding that such effects are difficult to maintain over the longer-term is largely consistent with several smaller trials published whilst PROPELS was ongoing. A physician-led physical activity intervention in 200 participants with established T2D reported a 6.8-min/day increase in moderate-to-vigorous physical activity after 12 months, but with effects reducing to 3.6 min after 36 months [23]; however, unlike PROPELS, there was evidence of sustained changes to auxiliary behaviours such as reductions to sedentary time and increases in light-intensity physical activity. The PACE-UP pedometer intervention for inactive adults demonstrated increases in ambulatory activity of between 600 and 700 steps/day over 36 months, but the effect for ambulatory activity was not sustained in 298 older adults aged 60–75 years over 48 months with differences in MVPA compared to control diminishing to 4.6 min/day [24]. The LookAHEAD lifestyle intervention for those with T2D reported that those in the intensive lifestyle intervention increased their MVPA by 8.3 min/day compared to baseline after 12 months, with the effect reducing to 1.9 min/day after 48 months [25]. Taken together, these results suggest that small, but nevertheless, potentially clinically meaningful, increases in physical activity are possible after receiving a behavioural intervention designed for inactive adults or those with metabolic dysfunction within family practice, but that such changes may be difficult for individuals to maintain into the longer-term. Longer-term physical activity and lifestyle intervention to date for the prevention and management of T2D have been based on individual-level behavioural interventions. However, factors like material and social deprivation and their impact on the physical environment are major determinants of health and health behaviour [26], including physical activity [27]. Therefore, it is possible that individual-level interventions may fail over the longer-term where the underlying socioeconomic determinants of physical inactive remain unchanged.

Although no longer-term changes in physical activity were reported, the Walking Away group lost weight and reduced their waist circumference by 1 kg and 1.6 cm compared to control at 48 months. Although sustained, these changes were relatively modest with smaller effects than interventions that are specifically aimed at long-term weight loss [28]. Whilst the impact of this degree of weight loss on mortality outcomes is uncertain [28], the Diabetes Prevention Program reported that each additional kilogramme of weight loss was associated with a 16% reduction in diabetes risk [29], suggesting this degree of weight loss may have conferred some cardiometabolic benefits to the Walking Away group. Interestingly, changes were not observed in the Walking Away Plus group, where markers of weight and adiposity were unchanged compared to control throughout the trial period. In Walking Away Plus, the mHealth follow-on support was specifically focused on physical activity only, which may have acted to dilute the dietary messages which were covered in the initial group-based intervention.

The key strengths of PROPELS are that it is the largest and longest physical activity trial in those with nondiabetic hyperglycaemia and it included a multi-ethnic family practice population and an objective measure of physical activity. Achieving the predefined target of at least 70% follow-up for objectively measured physical activity after 48 months is also a strength. However, there are potential limitations. The length and nature of the trial may have discouraged some potential participants from taking part, limiting generalisability. The relatively high levels of ambulatory activity and physical activity self-efficacy at baseline may have limited the effectiveness of the intervention at promoting further behaviour change. Objective measures of physical activity reduce error and bias but may exhibit Hawthorne-like effects (measurement reactivity), although these are believed to be minimal for MVPA among adults [30] and are mitigated further by having a control group. The degree of engagement with WAP (52% compliance with the per-protocol definition) may have limited the effectiveness of promoting maintained physical activity behaviour change. However, there was no evidence that physical activity behaviour change was maintained in those that achieved the per-protocol definition of adherence. The degree of adherence is consistent with previous implementation studies [31, 32], with data from the NHS Diabetes Prevention Programme reporting that approximately 60% of those that attended the initial assessment visit also attended at least one intervention session, with just over 10% completing the programme [4]. The PROPELS intervention was predominantly focused on increasing physical activity volume through walking behaviour. It is now increasingly recognised that reducing and breaking sedentary behaviour are also important behavioural targets for diabetes prevention and management that are independent of overall physical activity volume [33]. Future studies are therefore needed to investigate whether the integration of reduced sedentary behaviour goals into physical activity interventions more broadly can increase longer-term effectiveness. Finally, as participants were only followed up at 12 and 48 months, the trajectory of change between these time points was not evaluated, making it unclear whether a change in the WAP group was maintained beyond 12 months.

## Conclusions

In conclusion, the PROPELS study demonstrated that combining a pragmatic physical activity intervention with text messaging and telephone support results in modest changes in ambulatory activity over 12 months, but such changes were not maintained at 48 months. These findings, which are consistent with the wider literature, suggest individual-level behavioural interventions do not lead to clinically meaningful sustained increases in physical activity over the longer-term in high-risk groups.

## Supplementary Information

**Additional file 1:.** Characteristics of those with complete and missing primary outcome data by group.

**Additional file 2:.** Use of behaviour change techniques at follow-up.

**Additional file 3:.** Self-efficacy and illness perception scores at baseline and follow-up by group.

**Additional file 4:.** Per-protocol and multiple imputations results for the primary outcome.

**Additional file 5:.** Sub-group analysis testing whether intervention effect at 48-months for primary outcome is modified by key characteristics.

**Additional file 6:.** Baseline value with 12- and 48-month intervention effect for secondary outcomes.

**Additional file 7:.** Serious and non-serious adverse events.

## Data Availability

De-identified study data and supporting material (protocol, data dictionary and statistical analysis plan) will be shared 12 months after publication with researchers who provide a methodologically sound proposal and sign a data access agreement. Requests to access the data should be sent to the corresponding author.

## Abbreviations

ANCOVA: Analysis of covariance
BIPQ: Brief Illness Perceptions Questionnaire
EQ-5D-5L: European Quality of Life-5 Dimensions
EPIC: European Prospective Investigation of Cancer and Nutrition
HADS: Hospital Anxiety and Depression scale
IMD: Index of Multiple Deprivation
MVPA: Moderate-to-vigorous intensity physical activity
PROPELS: PRomotion Of Physical activity through structured Education with differing Levels of ongoing Support
T2D: Type 2 diabetes
WA: Walking Away
WAP: Walking Away Plus

## References

[1] Gillies CL, Abrams KR, Lambert PC, Cooper NJ, Sutton AJ, Hsu RT, Khunti K (2007). Pharmacological and lifestyle interventions to prevent or delay type 2 diabetes in people with impaired glucose tolerance: systematic review and meta-analysis. BMJ.

[2] Dunkley AJ, Bodicoat DH, Greaves CJ, Russell C, Yates T, Davies MJ, Khunti K (2014). Diabetes prevention in the real world: effectiveness of pragmatic lifestyle interventions for the prevention of type 2 diabetes and of the impact of adherence to guideline recommendations: a systematic review and meta-analysis. Diabetes Care.

[3] Ali MK, Bullard KM, Imperatore G, Benoit SR, Rolka DB, Albright AL (2019). Reach and use of diabetes prevention services in the United States, 2016-2017. JAMA Netw Open.

[4] Valabhji J, Barron E, Bradley D, Bakhai C, Fagg J, O’Neill S, Young B, Wareham N, Khunti K, Jebb S, Smith J (2020). Early Outcomes From the English National Health Service Diabetes Prevention Programme. Diabetes Care.

[5] Yates T, Khunti K, Bull F, Gorely T, Davies MJ (2007). The role of physical activity in the management of impaired glucose tolerance: a systematic review. Diabetologia.

[6] McCarthy M, Edwardson CL, Davies MJ, Henson J, Gray L, Khunti K (2017). Change in Sedentary Time, Physical Activity, Bodyweight, and Hba1c in High-Risk Adults. Med Sci Sports Exerc.

[7] Yates T, Haffner SM, Schulte PJ, Thomas L, Huffman KM, Bales CW, Califf RM, Holman RR, McMurray JJV, Bethel MA, Tuomilehto J, Davies MJ, Kraus WE (2014). Association between change in daily ambulatory activity and cardiovascular events in people with impaired glucose tolerance (NAVIGATOR trial): a cohort analysis. Lancet.

[8] Yates T, Edwardson CL, Henson J, Gray LJ, Ashra NB, Troughton J, Khunti K, Davies MJ (2017). Walking Away from Type 2 diabetes: a cluster randomized controlled trial. Diab Med.

[9] Yates T, Griffin S, Bodicoat DH, Brierly G, Dallosso H, Davies MJ, Eborall H, Edwardson C, Gillett M, Gray L, Hardeman W, Hill S, Morton K, Sutton S, Troughton J, Khunti K (2015). PRomotion Of Physical activity through structured Education with differing Levels of ongoing Support for people at high risk of type 2 diabetes (PROPELS): study protocol for a randomized controlled trial. Trials.

[10] National Institute for Health and Clinical Excellence. Preventing type 2 diabetes: risk identification and interventions for individuals at high risk. 2012; Available at: https://www.nice.org.uk/guidance/ph 38. Accessed 02/08, 2016.

[11] Morton KL, Sutton SR, Hardeman W, Troughton J, Yates T, Griffin SJ, et al. A text-messaging and pedometer program to promote physical activity in people at high risk of type 2 diabetes: A development and feasibility study for the PROPELS Trial. JMIR mHealth and uHealth. 2015;3(4):e105 10.2196/mhealth.5026 PMC4704921 26678750

[12] Lee JA, Williams SM, Brown DD, Laurson KR (2015). Concurrent validation of the Actigraph gt3x , Polar Active accelerometer, Omron HJ-720 and Yamax Digiwalker SW-701 pedometer step counts in lab-based and free-living settings. J Sports Sci.

[13] Abel MG, Peritore N, Shapiro R, Mullineaux DR, Rodriguez K, Hannon JC (2011). A comprehensive evaluation of motion sensor step-counting error. Appl Physiol Nutr Metab.

[14] Tudor-Locke C, Johnson WD, Katzmarzyk PT (2009). Accelerometer-determined steps per day in US adults. Med Sci Sports Exerc.

[15] Freedson PS, Melanson E, Sirard J (1998). Calibration of the Computer Science and Applications, Inc. accelerometer. Med Sci Sports Exerc.

[16] Besson H, Brage S, Jakes RW, Ekelund U, Wareham NJ (2009). Estimating physical activity energy expenditure, sedentary time, and physical activity intensity by self-report in adults. Am J Clin Nutr.

[17] Panter J, Jones A, van Sluijs E, Griffin S, Wareham N. Environmental and psychological correlates of older adult’s active commuting. Med Sci Sports Exerc 2011;43(7):1–17. 10.1249/MSS.0b013e3182078532 PMC3842528 21131863

[18] NAVIGATOR Study G, McMurray JJ, Holman RR, Haffner SM, Bethel MA, Holzhauer B, et al. (2010). Effect of valsartan on the incidence of diabetes and cardiovascular events. N Engl J Med.

[19] White IR, Carpenter J, Horton NJ (2012). Including all individuals is not enough: Lessons for intention-to-treat analysis. Clin Trials.

[20] Yates T, Gray LJ, Henson J, Edwardson CL, Khunti K, Davies MJ (2019). Impact of depression and anxiety on change to physical activity following a pragmatic diabetes prevention program within primary care: Pooled analysis from two randomized controlled trials. Diabetes Care.

[21] Chudasama YV, Khunti KK, Zaccardi F, Rowlands AV, Yates T, Gillies CL, Davies MJ, Dhalwani NN (2019). Physical activity, multimorbidity, and life expectancy: a UK Biobank longitudinal study. BMC Med.

[22] Rowlands A, Davies M, Dempsey P, Edwardson C, Razieh C, Yates T. Wrist-worn accelerometers: recommending~ 1.0 mg as the minimum clinically important difference (MCID) in daily average acceleration for inactive adults. Br J Sports Med. 2020. 10.1136/bjsports-2020-102293. 10.1136/bjsports-2020-102293 32928741

[23] Balducci S, D'Errico V, Haxhi J, Sacchetti M, Orlando G, Cardelli P (2019). Effect of a Behavioral Intervention Strategy on Sustained Change in Physical Activity and Sedentary Behavior in Patients With Type 2 Diabetes: The IDES_2 Randomized Clinical Trial. JAMA.

[24] Harris T, Kerry SM, Limb ES, Furness C, Wahlich C, Victor CR, et al. Physical activity levels in adults and older adults 3–4 years after pedometer-based walking interventions: Long-term follow-up of participants from two randomised controlled trials in UK primary care. Plos Med. 2018;15(3):1–16. 10.1371/journal.pmed.1002526 PMC5844512 29522529

[25] Unick JL, Gaussoin SA, Hill JO, Jakicic JM, Bond DS, Hellgren M (2016). Four-Year Physical Activity Levels among Intervention Participants with Type 2 Diabetes. Med Sci Sports Exerc.

[26] M Marmot M. Fair society, healthy lives: the Marmot review; strategic review of health inequalities in England post-2010. The Marmot Review, Institute for Health Equity, 2010. Available at: http://www.instituteofhealthequity.org/resources-reports/fair-society-healthy-lives-the-marmot-review (10 March 2021, date last accessed)

[27] Smith M, Hosking J, Woodward A, Witten K, MacMillan A, Field A (2017). Systematic literature review of built environment effects on physical activity and active transport–an update and new findings on health equity. Int J Beha Nutr Phys Activity.

[28] Singh N, Stewart RAH, Benatar JR (2019). Intensity and duration of lifestyle interventions for long-term weight loss and association with mortality: a meta-analysis of randomised trials. BMJ Open.

[29] Hamman RF, Wing RR, Edelstein SL, Lachin JM, Bray GA, Delahanty L, Hoskin M, Kriska AM, Mayer-Davis EJ, Pi-Sunyer X, Regensteiner J, Venditti B, Wylie-Rosett J, for the Diabetes Prevention Program Research Group (2006). Effect of weight loss with lifestyle intervention on risk of diabetes. Diabetes Care.

[30] Baumann S, Groß S, Voigt L, Ullrich A, Weymar F, Schwaneberg T, Dörr M, Meyer C, John U, Ulbricht S (2018). Pitfalls in accelerometer-based measurement of physical activity: The presence of reactivity in an adult population. Scand J Med Sci Sports.

[31] Ackermann RT, Finch EA, Brizendine E, Zhou H, Marrero DG (2008). Translating the Diabetes Prevention Program into the community: the DEPLOY pilot study. Am J Prev Med.

[32] Absetz P, Valve R, Oldenburg B, Heinonen H, Nissinen A, Fogelholm M, Ilvesmaki V, Talja M, Uutela A (2007). Type 2 diabetes prevention in the “real world”: one-year results of the GOAL Implementation Trial. Diabetes Care.

[33] Henson J, Dunstan DW, Davies MJ, Yates T (2016). Sedentary behaviour as a new behavioural target in the prevention and treatment of type 2 diabetes. Diab Metab Res Rev..

