# Supplementary material for: Promoting physical activity in a multi-ethnic population at high risk of diabetes: the 48-month PROPELS randomised controlled trial
Source: BMC Med. 2021 Jun 3;19:130. doi: 10.1186/s12916-021-01997-4 (PMC8173914; doi:10.1186/s12916-021-01997-4)
Supplement: Supplementary file 1 — Additional file 1:. Characteristics of those with complete and missing primary outcome data by group. [file 12916_2021_1997_MOESM1_ESM.docx]

# **Additional file 1: Characteristics of those with complete and missing primary outcome data by group**

##

| CONTROL GROUP |  |  |  |  |  |  |
| --- | --- | --- | --- | --- | --- | --- |
|  | **Complete data** | | | **Missing data** | | |
|  | **N=373** | | | **N=87** | | |
|  | **n** | **Mean** | **SD** | **n** | **Mean** | **SD** |
| Age (yrs) | 373 | 59.3 | 8.7 | 87 | 59.7 | 9.1 |
| BMI (kg/m^2^) | 373 | 29.3 | 5.9 | 87 | 29.6 | 5.1 |
| Social deprivation (IMD decile) | 373 | 5.5 | 2.8 | 86 | 5.5 | 3.0 |
|  |  | **%** | **n** |  | **%** | **n** |
| Sex | 373 |  |  | 87 |  |  |
| Men |  | 52.8 | 197 |  | 42.5 | 37 |
| Women |  | 47.2 | 176 |  | 57.5 | 50 |
| Ethnicity | 373 |  |  | 87 |  |  |
| White European |  | 70.2 | 262 |  | 74.7 | 65 |
| South Asian |  | 23.1 | 86 |  | 19.5 | 17 |
| Other |  | 6.7 | 25 |  | 5.7 | 5 |
| Family history of diabetes in first degree relatives | 370 | 43.2 | 160 | 87 | 43.7 | 38 |
| Prediabetes | 371 | 37.2 | 138 | 87 | 39.1 | 34 |
| Antihypertensive medication | 362 | 42.5 | 154 | 51 | 29.4 | 15 |
| Lipid lowering medication | 362 | 35.6 | 129 | 51 | 29.4 | 15 |
| Steroids | 373 | 5.9 | 22 | 87 | 13.8 | 12 |
| Metformin | 373 | 0.0 | 0 | 87 | 0.0 | 0 |
| CVD (MI, heart failure, angina, stroke) | 369 | 7.9 | 29 | 87 | 11.5 | 10 |
| Smoking status | 373 |  |  | 87 |  |  |
| Past |  | 35.7 | 133 |  | 49.4 | 43 |
| Current |  | 9.9 | 37 |  | 9.2 | 8 |
| Employment type | 373 |  |  | 87 |  |  |
| Full time |  | 39.4 | 147 |  | 29.9 | 26 |
| Part time |  | 15.3 | 57 |  | 19.5 | 17 |
| Retired |  | 34.3 | 128 |  | 37.9 | 33 |
| Unemployed or other |  | 11.0 | 41 |  | 12.6 | 11 |
| Educational status | 365 |  |  | 84 |  |  |
| Degree, higher degree or equivalent |  | 46.6 | 170 |  | 41.7 | 35 |
| Marital status | 373 |  |  | 87 |  |  |
| Married / civil partner |  | 69.4 | 259 |  | 63.2 | 55 |
| Access to the internet | 373 | 83.4 | 311 | 85 | 81.2 | 69 |

| WALKING AWAY GROUP |  |  |  |  |  |  |
| --- | --- | --- | --- | --- | --- | --- |
|  | **Complete data** | | | **Missing data** | | |
|  | **N=303** | | | **N=147** | | |
|  | **n** | **Mean** | **SD** | **n** | **Mean** | **SD** |
| Age (yrs) | 303 | 59.8 | 9.0 | 147 | 58.7 | 10.1 |
| BMI (kg/m^2^) | 303 | 28.9 | 5.1 | 147 | 29.6 | 6.3 |
| Social deprivation (IMD decile) | 302 | 6.0 | 3.0 | 147 | 5.1 | 3.0 |
|  |  | **%** | **n** |  | **%** | **n** |
| Sex | 303 |  |  | 147 |  |  |
| Men |  | 52.8 | 160 |  | 45.6 | 67 |
| Women |  | 47.2 | 143 |  | 54.4 | 80 |
| Ethnicity | 303 |  |  | 147 |  |  |
| White European |  | 73.9 | 224 |  | 69.4 | 102 |
| South Asian |  | 20.1 | 61 |  | 25.9 | 38 |
| Other |  | 5.9 | 18 |  | 4.8 | 7 |
| Family history of diabetes in first degree relatives | 301 | 42.5 | 128 | 147 | 40.8 | 60 |
| Prediabetes | 302 | 41.1 | 124 | 147 | 37.4 | 55 |
| Antihypertensive medication | 286 | 42.7 | 122 | 82 | 51.2 | 42 |
| Lipid lowering medication | 286 | 33.9 | 97 | 82 | 48.8 | 40 |
| Steroids | 303 | 8.3 | 25 | 147 | 10.9 | 16 |
| Metformin | 303 | 0.0 | 0 | 147 | 0.7 | 1 |
| CVD (MI, heart failure, angina, stroke) | 299 | 9.0 | 27 | 147 | 8.8 | 13 |
| Smoking status | 303 |  |  | 147 |  |  |
| Past |  | 36.3 | 110 |  | 36.1 | 53 |
| Current |  | 6.6 | 20 |  | 12.2 | 18 |
| Employment type | 303 |  |  | 147 |  |  |
| Full time |  | 33.7 | 102 |  | 35.4 | 52 |
| Part time |  | 20.8 | 63 |  | 19.7 | 29 |
| Retired |  | 37.0 | 112 |  | 32.0 | 47 |
| Unemployed or other |  | 8.6 | 26 |  | 12.9 | 19 |
| Educational status | 292 |  |  | 141 |  |  |
| Degree, higher degree or equivalent |  | 48.3 | 141 |  | 39.7 | 56 |
| Marital status | 303 |  |  | 147 |  |  |
| Married / civil partner |  | 76.6 | 232 |  | 73.5 | 108 |
| Access to the internet | 302 | 89.1 | 269 | 147 | 80.3 | 118 |

| WALKING AWAY PLUS GROUP |  |  |  |  |  |  |
| --- | --- | --- | --- | --- | --- | --- |
|  | **Complete data** | | | **Missing data** | | |
|  | **N=317** | | | **N=139** | | |
|  | **n** | **Mean** | **SD** | **n** | **Mean** | **SD** |
| Age (yrs) | 317 | 60.0 | 8.5 | 139 | 57.8 | 10.2 |
| BMI (kg/m^2^) | 317 | 29.0 | 5.5 | 139 | 29.7 | 5.8 |
| Social deprivation (IMD decile) | 317 | 5.9 | 2.7 | 139 | 5.2 | 2.9 |
|  |  | **%** | **n** |  | **%** | **n** |
| Sex | 317 |  |  | 139 |  |  |
| *Men* |  | 48.6 | 154 |  | 56.1 | 78 |
| *Women* |  | 51.4 | 163 |  | 43.9 | 61 |
| Ethnicity | 317 |  |  | 139 |  |  |
| *White European* |  | 74.4 | 236 |  | 66.9 | 93 |
| *South Asian* |  | 20.8 | 66 |  | 26.6 | 37 |
| *Other* |  | 4.7 | 15 |  | 6.5 | 9 |
| Family history of diabetes in first degree relatives | 316 | 44.3 | 140 | 137 | 47.4 | 65 |
| Prediabetes | 316 | 39.9 | 126 | 139 | 36.0 | 50 |
| Antihypertensive medication | 300 | 45.7 | 137 | 80 | 41.3 | 33 |
| Lipid lowering medication | 300 | 39.7 | 119 | 79 | 39.2 | 31 |
| Steroids | 317 | 6.0 | 19 | 139 | 7.2 | 10 |
| Metformin | 317 | 0.3 | 1 | 139 | 0.0 | 0 |
| CVD (MI, heart failure, angina, stroke) | 316 | 8.9 | 28 | 138 | 12.3 | 17 |
| Smoking status | 317 |  |  | 139 |  |  |
| Past |  | 39.7 | 126 |  | 34.5 | 48 |
| Current |  | 7.9 | 25 |  | 19.4 | 27 |
| Employment type | 317 |  |  | 139 |  |  |
| Full time |  | 36.9 | 117 |  | 37.4 | 52 |
| Part time |  | 19.2 | 61 |  | 18.0 | 25 |
| Retired |  | 35.3 | 112 |  | 29.5 | 41 |
| Unemployed or other |  | 8.5 | 27 |  | 15.1 | 21 |
| Educational status | 314 |  |  | 136 |  |  |
| Degree, higher degree or equivalent |  | 49.7 | 156 |  | 33.8 | 46 |
| Marital status | 317 |  |  | 139 |  |  |
| Married / civil partner |  | 77.0 | 244 |  | 66.9 | 93 |
| Access to the internet | 317 | 87.4 | 277 | 138 | 80.4 | 111 |
