# Supplementary material for: Promoting physical activity in a multi-ethnic population at high risk of diabetes: the 48-month PROPELS randomised controlled trial
Source: BMC Med. 2021 Jun 3;19:130. doi: 10.1186/s12916-021-01997-4 (PMC8173914; doi:10.1186/s12916-021-01997-4)
Supplement: Supplementary file 2 — Additional file 2:. Use of behaviour change techniques at follow-up. [file 12916_2021_1997_MOESM2_ESM.docx]

# **Additional file 2: Use of behaviour change techniques at follow-up**

|  | CONTROL | | | | Walking Away | | | | Walking Away Plus | | | | | |  |
| --- | --- | --- | --- | --- | --- | --- | --- | --- | --- | --- | --- | --- | --- | --- | --- |
|  | **12 months** | | **48 months** | | **12 months** | | **48 months** | | **12 months** | | | **48 months** | | |  |
|  | **%** | **n** | **%** | **n** | **%** | **n** | **%** | **n** | | **%** | **n** | | **%** | **n** | |
| Set regular goals detailing amount of exercise you would do each day | | | | | | | | | | | | | | |  |
| Most of the time | 30.0 | 119 | 30.6 | 116 | 34.2 | 118 | 39.0 | 123 | | 40.3 | 139 | | 38.5 | 125 | |
| Some of the time | 34.0 | 135 | 31.1 | 118 | 37.4 | 129 | 34.0 | 107 | | 38.6 | 133 | | 40.3 | 131 | |
| Rarely | 17.9 | 71 | 20.8 | 79 | 18.3 | 63 | 16.8 | 53 | | 13.9 | 48 | | 12.0 | 39 | |
| Never | 18.1 | 72 | 17.4 | 66 | 10.1 | 35 | 10.2 | 32 | | 7.2 | 25 | | 9.2 | 30 | |
| Regularly set plan detailing where, when and how you would exercise | | | | | | | | | | | | | | |  |
| Most of the time | 25.4 | 101 | 25.6 | 97 | 25.9 | 89 | 34.6 | 109 | | 27.9 | 96 | | 30.8 | 100 | |
| Some of the time | 31.0 | 123 | 30.3 | 115 | 38.1 | 131 | 31.7 | 100 | | 43.9 | 151 | | 36.3 | 118 | |
| Rarely | 21.2 | 84 | 22.4 | 85 | 23.8 | 82 | 22.5 | 71 | | 18.0 | 62 | | 22.8 | 74 | |
| Never | 22.4 | 89 | 21.6 | 82 | 12.2 | 42 | 11.1 | 35 | | 10.2 | 35 | | 10.2 | 33 | |
| Worn a pedometer | | | | | | | | | | | | | | |  |
| Most of the time | 2.5 | 10 | 9.0 | 34 | 22.7 | 78 | 25.2 | 79 | | 36.4 | 126 | | 32.4 | 105 | |
| Some of the time | 10.1 | 40 | 10.6 | 40 | 28.5 | 98 | 24.5 | 77 | | 36.7 | 127 | | 31.8 | 103 | |
| Rarely | 11.9 | 47 | 13.6 | 51 | 26.2 | 90 | 17.8 | 56 | | 15.0 | 52 | | 18.5 | 60 | |
| Never | 75.4 | 298 | 66.8 | 251 | 22.7 | 78 | 32.5 | 102 | | 11.8 | 41 | | 17.3 | 56 | |
| Kept an exercise log recording your activity levels | | | | | | | | | | | | | | |  |
| Most of the time | 4.8 | 19 | 7.7 | 29 | 17.1 | 59 | 15.6 | 49 | | 24.1 | 83 | | 19.7 | 64 | |
| Some of the time | 6.3 | 25 | 9.8 | 37 | 18.0 | 62 | 15.0 | 47 | | 30.2 | 104 | | 21.2 | 69 | |
| Rarely | 8.3 | 33 | 11.9 | 45 | 17.7 | 61 | 24.2 | 76 | | 22.4 | 77 | | 23.7 | 77 | |
| Never | 80.6 | 319 | 70.7 | 268 | 47.2 | 163 | 45.2 | 142 | | 23.3 | 80 | | 35.4 | 115 | |
| Been aware of your activity levels | | | | | | | | | | | | | | |  |
| Most of the time | 41.1 | 163 | 40.1 | 152 | 55.8 | 193 | 61.9 | 195 | | 66.7 | 230 | | 64.6 | 210 | |
| Some of the time | 30.0 | 119 | 34.8 | 132 | 26.9 | 93 | 22.2 | 70 | | 24.9 | 86 | | 24.9 | 81 | |
| Rarely | 12.1 | 48 | 11.1 | 42 | 9.8 | 34 | 9.8 | 31 | | 4.9 | 17 | | 5.2 | 17 | |
| Never | 16.9 | 67 | 14.0 | 53 | 7.5 | 26 | 6.0 | 19 | | 3.5 | 12 | | 5.2 | 17 | |
| Tried to exercise regularly | | | | | | | | | | | | | | |  |
| Most of the time | 41.8 | 166 | 42.0 | 159 | 53.0 | 183 | 53.8 | 169 | | 56.1 | 194 | | 56.6 | 184 | |
| Some of the time | 35.0 | 139 | 32.7 | 124 | 34.8 | 120 | 31.2 | 98 | | 33.5 | 116 | | 32.9 | 107 | |
| Rarely | 13.6 | 54 | 15.3 | 58 | 7.5 | 26 | 10.2 | 32 | | 8.4 | 29 | | 7.1 | 23 | |
| Never | 9.6 | 38 | 10.0 | 38 | 4.6 | 16 | 4.8 | 15 | | 2.0 | 7 | | 3.4 | 11 | |
