# Supplementary material for: Promoting physical activity in a multi-ethnic population at high risk of diabetes: the 48-month PROPELS randomised controlled trial
Source: BMC Med. 2021 Jun 3;19:130. doi: 10.1186/s12916-021-01997-4 (PMC8173914; doi:10.1186/s12916-021-01997-4)
Supplement: Supplementary file 3 — Additional file 3:. Self-efficacy and illness perception scores at baseline and follow-up by group. [file 12916_2021_1997_MOESM3_ESM.docx]

# **Additional file 3: Self-efficacy and illness perception scores at baseline and follow-up by group**

|  | CONTROL | | | | | | | | | | | |  |
| --- | --- | --- | --- | --- | --- | --- | --- | --- | --- | --- | --- | --- | --- |
|  | **Baseline** | | | | **12 months** | | | | **48 months** | | | |  |
|  | **N** | **Median** | **p25** | **p75** | **N** | **Median** | **p25** | **p75** | **N** | **Median** | **p25** | **p75** | |
| **Walking self-efficacy** |  |  |  |  |  |  |  |  |  |  |  |  | |
| Confidence to walk for 10 minutes per day (%) | 459 | 100 | 100 | 100 | 397 | 100 | 100 | 100 | 379 | 100 | 100 | 100 | |
| Confidence to walk for 30 minutes per day (%) | 458 | 100 | 80 | 100 | 397 | 100 | 80 | 100 | 379 | 100 | 90 | 100 | |
| Confidence to walk for 60 minutes per day (%) | 459 | 90 | 50 | 100 | 397 | 90 | 50 | 100 | 379 | 90 | 50 | 100 | |
| **Illness perception** |  |  |  |  |  |  |  |  |  |  |  |  | |
| How much does your risk of diabetes affect your life? | 310 | 4.0 | 1.0 | 7.0 | 395 | 4.0 | 1.0 | 6.0 | 379 | 4.0 | 1.0 | 6.0 | |
| How long do you think your risk of diabetes will continue? | 310 | 5.0 | 3.0 | 10.0 | 393 | 7.0 | 4.0 | 10.0 | 380 | 7.0 | 3.0 | 10.0 | |
| How much control do you feel you have over your risk of diabetes? | 310 | 7.0 | 5.0 | 8.0 | 394 | 7.0 | 5.0 | 8.0 | 380 | 7.0 | 5.0 | 8.0 | |
| How much do you think treatment can help your risk of diabetes? | 310 | 8.0 | 6.0 | 10.0 | 393 | 8.0 | 5.0 | 10.0 | 378 | 7.0 | 5.0 | 9.0 | |
| How much do you experience symptoms from your risk of diabetes? | 312 | 0.0 | 0.0 | 2.0 | 394 | 0.0 | 0.0 | 2.0 | 379 | 0.0 | 0.0 | 2.0 | |
| How concerned are you about your risk of diabetes? | 311 | 7.0 | 3.0 | 9.0 | 396 | 6.0 | 3.0 | 9.0 | 380 | 6.0 | 3.0 | 8.0 | |
| How well do you feel you understand your risk of diabetes? | 312 | 7.0 | 4.5 | 9.0 | 394 | 7.0 | 5.0 | 10.0 | 380 | 8.0 | 5.0 | 10.0 | |
| How much does your risk of diabetes affect you emotionally? | 312 | 3.0 | 0.0 | 6.0 | 394 | 2.0 | 0.0 | 5.0 | 380 | 2.0 | 0.0 | 5.0 | |

|  | WALKING AWAY | | | | | | | | | | | |  |
| --- | --- | --- | --- | --- | --- | --- | --- | --- | --- | --- | --- | --- | --- |
|  | **Baseline** | | | | **12 months** | | | | **48 months** | | | |  |
|  | **N** | **Median** | **p25** | **p75** | **N** | **Median** | **p25** | **p75** | **N** | **Median** | **p25** | **p75** | |
| **Walking self-efficacy** |  |  |  |  |  |  |  |  |  |  |  |  | |
| Confidence to walk for 10 minutes per day (%) | 450 | 100 | 100 | 100 | 347 | 100 | 100 | 100 | 315 | 100 | 100 | 100 | |
| Confidence to walk for 30 minutes per day (%) | 450 | 100 | 80 | 100 | 346 | 100 | 90 | 100 | 315 | 100 | 80 | 100 | |
| Confidence to walk for 60 minutes per day (%) | 449 | 90 | 50 | 100 | 346 | 90 | 50 | 100 | 315 | 100 | 50 | 100 | |
| **Illness perception** |  |  |  |  |  |  |  |  |  |  |  |  | |
| How much does your risk of diabetes affect your life? | 303 | 3.0 | 1.0 | 6.0 | 346 | 3.0 | 1.0 | 6.0 | 315 | 5.0 | 1.0 | 7.0 | |
| How long do you think your risk of diabetes will continue? | 303 | 6.0 | 3.0 | 10.0 | 344 | 7.0 | 4.0 | 10.0 | 314 | 7.0 | 3.0 | 10.0 | |
| How much control do you feel you have over your risk of diabetes? | 303 | 7.0 | 5.0 | 8.0 | 344 | 7.0 | 5.0 | 8.0 | 315 | 7.0 | 6.0 | 9.0 | |
| How much do you think treatment can help your risk of diabetes? | 303 | 8.0 | 6.0 | 10.0 | 342 | 7.0 | 5.0 | 9.0 | 314 | 8.0 | 5.0 | 10.0 | |
| How much do you experience symptoms from your risk of diabetes? | 301 | 0.0 | 0.0 | 2.0 | 346 | 0.0 | 0.0 | 2.0 | 315 | 0.0 | 0.0 | 3.0 | |
| How concerned are you about your risk of diabetes? | 302 | 7.0 | 4.0 | 10.0 | 347 | 7.0 | 4.0 | 9.0 | 312 | 7.0 | 4.0 | 9.0 | |
| How well do you feel you understand your risk of diabetes? | 302 | 7.0 | 5.0 | 9.0 | 345 | 8.0 | 6.0 | 10.0 | 313 | 9.0 | 7.0 | 10.0 | |
| How much does your risk of diabetes affect you emotionally? | 302 | 3.0 | 0.0 | 5.0 | 347 | 2.0 | 0.0 | 5.0 | 314 | 2.0 | 0.0 | 5.0 | |

|  | WALKING AWAY PLUS | | | | | | | | | | | |  |
| --- | --- | --- | --- | --- | --- | --- | --- | --- | --- | --- | --- | --- | --- |
|  | **Baseline** | | | | **12 months** | | | | **48 months** | | | |  |
|  | **N** | **Median** | **p25** | **p75** | **N** | **Median** | **p25** | **p75** | **N** | **Median** | **p25** | **p75** | |
| **Walking self-efficacy** |  |  |  |  |  |  |  |  |  |  |  |  | |
| Confidence to walk for 10 minutes per day (%) | 455 | 100 | 100 | 100 | 346 | 100 | 100 | 100 | 324 | 100 | 100 | 100 | |
| Confidence to walk for 30 minutes per day (%) | 455 | 100 | 80 | 100 | 345 | 100 | 90 | 100 | 324 | 100 | 80 | 100 | |
| Confidence to walk for 60 minutes per day (%) | 452 | 100 | 50 | 100 | 345 | 100 | 50 | 100 | 324 | 95 | 50 | 100 | |
| **Illness perception** |  |  |  |  |  |  |  |  |  |  |  |  | |
| How much does your risk of diabetes affect your life? | 308 | 3.0 | 1.0 | 5.0 | 342 | 4.0 | 1.0 | 7.0 | 325 | 5.0 | 1.0 | 8.0 | |
| How long do you think your risk of diabetes will continue? | 309 | 5.0 | 3.0 | 10.0 | 342 | 7.0 | 4.0 | 10.0 | 323 | 8.0 | 5.0 | 10.0 | |
| How much control do you feel you have over your risk of diabetes? | 310 | 6.0 | 5.0 | 8.0 | 342 | 7.0 | 5.0 | 8.0 | 325 | 7.0 | 5.0 | 9.0 | |
| How much do you think treatment can help your risk of diabetes? | 309 | 8.0 | 6.0 | 10.0 | 341 | 8.0 | 6.0 | 10.0 | 325 | 7.0 | 5.0 | 9.0 | |
| How much do you experience symptoms from your risk of diabetes? | 308 | 0.0 | 0.0 | 2.0 | 341 | 0.0 | 0.0 | 2.0 | 324 | 0.0 | 0.0 | 2.0 | |
| How concerned are you about your risk of diabetes? | 308 | 7.0 | 4.0 | 9.5 | 342 | 7.0 | 4.0 | 10.0 | 325 | 7.0 | 5.0 | 9.0 | |
| How well do you feel you understand your risk of diabetes? | 310 | 6.0 | 4.0 | 9.0 | 343 | 8.0 | 7.0 | 10.0 | 325 | 9.0 | 7.0 | 10.0 | |
| How much does your risk of diabetes affect you emotionally? | 309 | 3.0 | 0.0 | 5.0 | 342 | 2.0 | 0.0 | 5.0 | 325 | 2.0 | 0.0 | 5.0 | |
