# Supplementary material for: Promoting physical activity in a multi-ethnic population at high risk of diabetes: the 48-month PROPELS randomised controlled trial
Source: BMC Med. 2021 Jun 3;19:130. doi: 10.1186/s12916-021-01997-4 (PMC8173914; doi:10.1186/s12916-021-01997-4)
Supplement: Supplementary file 4 — Additional file 4:. Per-protocol and multiple imputations results for the primary outcome. [file 12916_2021_1997_MOESM4_ESM.docx]

# **Additional file 4: Per-protocol and multiple imputations results for the primary outcome**

|  |  | Walking Away vs Control at 48 months | | | Walking Away Plus vs Control at 48 months | | |
| --- | --- | --- | --- | --- | --- | --- | --- |
|  | Model N | Difference | 97.5% CI lower | 97.5% CI upper | Difference | 97.5% CI lower | 97.5% CI upper |
| Per-protocol | 824 | 29.9 | -359.2 | 418.9 | 426.9 | -62.7 | 916.4 |
| Multiple Imputation | 1366 | 87.6 | -308.2 | 483.3 | 183.7 | -258.4 | 625.8 |
