# Supplementary material for: Promoting physical activity in a multi-ethnic population at high risk of diabetes: the 48-month PROPELS randomised controlled trial
Source: BMC Med. 2021 Jun 3;19:130. doi: 10.1186/s12916-021-01997-4 (PMC8173914; doi:10.1186/s12916-021-01997-4)
Supplement: Supplementary file 5 — Additional file 5:. Sub-group analysis testing whether intervention effect at 48-months for primary outcome is modified by key characteristics. [file 12916_2021_1997_MOESM5_ESM.docx]

| **Factor tested** | **p-value for interaction** |
| --- | --- |
| Sex (men / women) | 0.321 |
| Age (<60 years / ≥60 years) | 0.420 |
| Ethnicity (White European / South Asian / Other) | 0.114 |
| Family history of T2D (no / yes) | 0.977 |
| Prediabetes at baseline (no / yes) | 0.474 |
| Baseline obesity status (<30kg/m^2^ / ≥30 kg/m^2^ in White Europeans/other; <27.5kg/m^2^ / ≥27.5 kg/m^2^ in South Asians) | 0.734 |
| Baseline deprivation (below / above median IMD decile) | 0.035 |

# **Additional file 5: Sub-group analysis testing whether the intervention effect at 48-months for the primary outcome is modified by key characteristics**

**Stratified analysis for significant interactions**

|  | Walking Away vs Control at 48 months | | | Walking Away Plus vs Control at 48 months | | |
| --- | --- | --- | --- | --- | --- | --- |
|  | Difference | 97.5% CI lower | 97.5% CI upper | Difference | 97.5% CI lower | 97.5% CI upper |
| High social deprivation (below the median IMD decile) | -46.1 | -584.3 | 492.1 | -369.7 | -944.8 | 205.4 |
| Low social deprivation  (at or above the median IMD decile) | 170.0 | -342.7 | 682.7 | 479.6 | -73.3 | 1032.5 |
