# Supplementary material for: Promoting physical activity in a multi-ethnic population at high risk of diabetes: the 48-month PROPELS randomised controlled trial
Source: BMC Med. 2021 Jun 3;19:130. doi: 10.1186/s12916-021-01997-4 (PMC8173914; doi:10.1186/s12916-021-01997-4)
Supplement: Supplementary file 6 — Additional file 6:. Baseline value with 12- and 48-month intervention effect for secondary outcomes. [file 12916_2021_1997_MOESM6_ESM.docx]

# **Additional file 6: Baseline value with 12-month and 48-month intervention effect for secondary outcomes**

|  | Control | | Walking Away | | Walking Away Plus | | Walking Away vs control | | | Walking Away Plus vs Control | | | Walking Away vs Control | | | Walking Away Plus vs Control | | |
| --- | --- | --- | --- | --- | --- | --- | --- | --- | --- | --- | --- | --- | --- | --- | --- | --- | --- | --- |
|  | Baseline | | | | | | 12 month intervention effect | | | | | | 48 month intervention effect | | | | | |
|  | Mean | SD | Mean | SD | Mean | SD | Difference | 97.5% CI lower | 97.5% CI upper | Difference | 97.5% CI lower | 97.5% CI upper | Difference | 97.5% CI lower | | Difference | 97.5% CI upper | |
| Self-reported physical activity | | | | | |  |  |  |  |  |  |  |  |  |  |  |  |  |
| Overall physical activity expenditure (kJ/kg/day) | 33.5 | 38.4 | 32.9 | 33.3 | 30.2 | 32.4 | 0.3 | -3.9 | 4.4 | 2.5 | -1.9 | 6.8 | 2.3 | -2.3 | 6.9 | 4.4 | 0.0 | 8.8 |
| Main biochemistry outcomes | | | |  |  |  |  |  |  |  |  |  |  |  |  |  |  |  |
| HbA1c (mmol/mol) | 40.0 | 3.7 | 40.5 | 3.5 | 40.4 | 3.5 | -0.14 | -0.47 | 0.20 | -0.10 | -0.43 | 0.23 | -0.13 | -0.68 | 0.42 | -0.01 | -0.63 | 0.61 |
| HbA1c (%) | 5.8 | 0.3 | 5.9 | 0.3 | 5.9 | 0.3 | -0.02 | -0.05 | 0.01 | -0.01 | -0.04 | 0.02 | -0.02 | -0.07 | 0.03 | -0.03 | -0.07 | 0.02 |
| Total cholesterol (mmol/l) | 5.2 | 1.1 | 5.2 | 1.1 | 5.2 | 1.1 | -0.04 | -0.15 | 0.06 | -0.08 | -0.18 | 0.03 | 0.02 | -0.11 | 0.15 | -0.02 | -0.16 | 0.11 |
| HDL cholesterol (mmol/l) | 1.4 | 0.4 | 1.4 | 0.4 | 1.4 | 0.4 | 0.00 | -0.03 | 0.04 | 0.01 | -0.03 | 0.05 | 0.00 | -0.03 | 0.04 | 0.04 | -0.01 | 0.08 |
| LDL cholesterol (mmol/l) | 3.0 | 0.9 | 3.1 | 0.9 | 3.1 | 1.0 | -0.02 | -0.12 | 0.07 | -0.04 | -0.13 | 0.05 | 0.03 | -0.08 | 0.15 | 0.00 | -0.11 | 0.12 |
| Triglycerides (mmol/l) | 1.5 | 0.8 | 1.6 | 1.0 | 1.5 | 0.8 | -0.09 | -0.25 | 0.06 | -0.15 | -0.29 | -0.01 | -0.07 | -0.18 | 0.03 | -0.11 | -0.21 | 0.00 |
| Vitamin D (nmol/l) | 45.8 | 23.1 | 44.1 | 20.9 | 43.8 | 22.6 | 1.52 | -2.50 | 5.53 | 0.42 | -3.20 | 4.04 | 1.17 | -2.79 | 5.12 | 1.63 | -2.26 | 5.52 |
| Other biochemistry outcomes | | | |  |  |  |  |  |  |  |  |  |  |  |  |  |  |  |
| Sodium (mmol/l) | 140.4 | 2.0 | 140.5 | 2.0 | 140.3 | 2.1 | -0.12 | -0.39 | 0.15 | 0.03 | -0.24 | 0.29 | -0.06 | -0.38 | 0.25 | 0.09 | -0.25 | 0.42 |
| Potassium (mmol/l) | 4.3 | 0.4 | 4.3 | 0.4 | 4.3 | 0.4 | -0.03 | -0.09 | 0.02 | -0.04 | -0.10 | 0.01 | 0.02 | -0.04 | 0.08 | -0.03 | -0.09 | 0.02 |
| Urea (mmol/l) | 5.8 | 1.5 | 5.8 | 1.5 | 5.9 | 1.6 | 0.05 | -0.13 | 0.23 | -0.08 | -0.26 | 0.09 | 0.04 | -0.17 | 0.25 | -0.07 | -0.27 | 0.13 |
| eGFR (ml/min/1.73m^2^) | 85.6 | 19.9 | 85.5 | 12.1 | 85.5 | 12.6 | 0.29 | -1.64 | 2.23 | 0.26 | -1.72 | 2.23 | -0.40 | -2.36 | 1.56 | 0.42 | -1.54 | 2.38 |
| Total bilirubin (umol/l) | 10.3 | 5.0 | 10.3 | 5.1 | 10.5 | 5.5 | 0.17 | -0.36 | 0.70 | -0.11 | -0.61 | 0.40 | -0.09 | -0.60 | 0.42 | -0.19 | -0.77 | 0.38 |
| ALP (IU/l) | 82.7 | 22.0 | 82.8 | 23.5 | 79.8 | 22.5 | -0.85 | -2.87 | 1.18 | -1.32 | -3.10 | 0.46 | -3.70 | -6.45 | -0.96 | -1.08 | -3.65 | 1.49 |
| ALT (IU/l) | 26.3 | 13.0 | 26.6 | 13.4 | 26.8 | 16.1 | -1.33 | -2.92 | 0.26 | -0.89 | -2.40 | 0.62 | -1.79 | -3.51 | -0.07 | 1.55 | -1.99 | 5.08 |
| GGT (IU/l) | 34.7 | 31.4 | 35.1 | 34.3 | 37.3 | 42.6 | -2.93 | -6.60 | 0.74 | -1.99 | -5.24 | 1.26 | -4.67 | -12.35 | 3.00 | -3.87 | -11.47 | 3.72 |
| Urine albumin creatinine ratio (mg/mmol) | 1.5 | 3.3 | 1.3 | 2.9 | 1.4 | 3.8 | -0.29 | -0.95 | 0.38 | -0.09 | -0.77 | 0.60 | -0.10 | -1.11 | 0.91 | -0.77 | -1.70 | 0.17 |
| Cardiovascular risk | | | |  |  |  |  |  |  |  |  |  |  |  |  |  |  |  |
| 10-year cardiovascular risk (Framingham) (%) | 13.8 | 10.2 | 14.4 | 9.4 | 14.5 | 10.6 | -0.86 | -1.69 | -0.04 | -0.73 | -1.57 | 0.11 | -0.26 | -1.31 | 0.78 | -0.54 | -1.65 | 0.57 |
| Anthropometry |  |  |  |  |  |  |  |  |  |  |  |  |  |  |  |  |  |  |
| Weight (kg) | 82.3 | 17.8 | 81.2 | 17.7 | 81.7 | 18.6 | -0.60 | -1.18 | -0.03 | -0.05 | -0.62 | 0.52 | -1.00 | -1.92 | -0.07 | -0.23 | -1.16 | 0.70 |
| BMI (kg/m^2^) | 29.3 | 5.7 | 29.1 | 5.6 | 29.2 | 5.6 | -0.20 | -0.41 | 0.01 | -0.01 | -0.21 | 0.20 | -0.42 | -0.77 | -0.07 | -0.23 | -0.57 | 0.12 |
| Waist circumference (cm) | 98.9 | 13.6 | 98.7 | 13.9 | 98.8 | 14.4 | -1.28 | -2.18 | -0.38 | -0.47 | -1.39 | 0.45 | -1.57 | -2.70 | -0.45 | -1.09 | -2.33 | 0.15 |
| Body fat percentage (%) | 33.6 | 9.5 | 33.6 | 9.1 | 33.5 | 8.9 | -0.50 | -0.98 | -0.03 | -0.31 | -0.80 | 0.17 | -1.06 | -1.79 | -0.33 | -0.47 | -1.15 | 0.22 |
| Fat mass (kg) | 28.6 | 12.6 | 27.9 | 11.6 | 28.1 | 11.8 | -0.24 | -0.87 | 0.39 | 0.08 | -0.58 | 0.75 | -0.90 | -1.96 | 0.17 | -0.31 | -1.40 | 0.79 |
| Fat free mass (kg) | 53.5 | 10.8 | 53.2 | 11.1 | 53.6 | 11.6 | -0.40 | -0.93 | 0.14 | -0.06 | -0.61 | 0.49 | -0.72 | -1.61 | 0.17 | -0.66 | -1.55 | 0.24 |
| Depression and anxiety |  |  |  |  |  |  |  |  |  |  |  |  |  |  |  |  |  |  |
| Depression score | 4.0 | 3.3 | 3.7 | 3.2 | 4.2 | 3.7 | -0.21 | -0.56 | 0.13 | -0.34 | -0.70 | 0.02 | 0.05 | -0.37 | 0.47 | -0.09 | -0.54 | 0.37 |
| Anxiety score | 6.1 | 4.2 | 5.8 | 4.0 | 6.0 | 4.0 | -0.21 | -0.63 | 0.20 | -0.14 | -0.59 | 0.32 | 0.19 | -0.30 | 0.68 | -0.31 | -0.84 | 0.23 |
| Diet |  |  |  |  |  |  |  |  |  |  |  |  |  |  |  |  |  |  |
| Frequency (portions/week) |  |  |  |  |  |  |  |  |  |  |  |  |  |  |  |  |  |  |
| Fresh fruit | 4.7 | 1.3 | 4.8 | 1.3 | 4.8 | 1.3 | 0.23 | 0.07 | 0.39 | 0.12 | -0.04 | 0.29 | 0.22 | 0.05 | 0.40 | 0.13 | -0.04 | 0.30 |
| Green leafy vegetables | 4.3 | 1.2 | 4.5 | 1.1 | 4.4 | 1.2 | 0.12 | -0.03 | 0.26 | 0.14 | 0.00 | 0.29 | 0.24 | 0.07 | 0.41 | 0.24 | 0.08 | 0.41 |
| Other vegetables | 4.7 | 1.0 | 4.8 | 1.0 | 4.8 | 1.0 | 0.21 | 0.07 | 0.35 | 0.17 | 0.04 | 0.31 | 0.20 | 0.05 | 0.35 | 0.20 | 0.06 | 0.35 |
| Oily fish | 2.6 | 1.1 | 2.7 | 1.2 | 2.7 | 1.2 | 0.02 | -0.12 | 0.16 | 0.09 | -0.05 | 0.22 | 0.00 | -0.16 | 0.15 | 0.04 | -0.12 | 0.20 |
| Other fish | 2.7 | 1.0 | 2.7 | 1.0 | 2.7 | 1.0 | -0.06 | -0.20 | 0.08 | 0.03 | -0.11 | 0.16 | -0.02 | -0.17 | 0.14 | 0.06 | -0.10 | 0.22 |
| Chicken | 3.4 | 1.1 | 3.3 | 1.1 | 3.4 | 1.1 | 0.05 | -0.06 | 0.16 | 0.06 | -0.05 | 0.18 | -0.01 | -0.16 | 0.13 | 0.02 | -0.12 | 0.15 |
| Meat | 3.1 | 1.2 | 3.2 | 1.2 | 3.3 | 1.2 | -0.02 | -0.15 | 0.11 | 0.00 | -0.14 | 0.14 | -0.14 | -0.29 | 0.00 | -0.12 | -0.27 | 0.03 |
| Eggs | 3.3 | 1.1 | 3.4 | 1.2 | 3.4 | 1.1 | -0.02 | -0.15 | 0.11 | -0.01 | -0.14 | 0.12 | 0.07 | -0.09 | 0.23 | 0.05 | -0.11 | 0.21 |
| Cheese | 3.6 | 1.2 | 3.6 | 1.1 | 3.7 | 1.2 | -0.15 | -0.30 | 0.00 | -0.16 | -0.31 | -0.01 | 0.00 | -0.16 | 0.17 | -0.06 | -0.24 | 0.11 |
| Wholemeal/brown bread | 4.1 | 1.6 | 4.2 | 1.6 | 4.3 | 1.6 | 0.05 | -0.17 | 0.26 | 0.03 | -0.19 | 0.25 | 0.07 | -0.17 | 0.31 | 0.06 | -0.18 | 0.30 |
| Alcohol (drinks/day) | 2.0 | 0.9 | 1.9 | 0.8 | 2.0 | 0.8 | -0.01 | -0.09 | 0.08 | -0.02 | -0.11 | 0.07 | 0.01 | -0.09 | 0.10 | -0.01 | -0.10 | 0.09 |
| Number of days/week on which individual reported limiting intake of: | | | | | | | | |  |  |  |  |  |  |  |  |  |  |
| Total fat | 4.4 | 1.6 | 4.5 | 1.5 | 4.5 | 1.6 | 0.11 | -0.11 | 0.32 | 0.09 | -0.12 | 0.31 | 0.32 | 0.09 | 0.55 | 0.32 | 0.09 | 0.55 |
| Saturated fat | 4.5 | 1.7 | 4.5 | 1.6 | 4.6 | 1.6 | 0.17 | -0.06 | 0.39 | 0.08 | -0.14 | 0.31 | 0.41 | 0.18 | 0.65 | 0.37 | 0.12 | 0.61 |
| Sugar | 4.7 | 1.7 | 4.7 | 1.5 | 4.7 | 1.5 | 0.09 | -0.11 | 0.29 | 0.06 | -0.17 | 0.29 | 0.34 | 0.12 | 0.55 | 0.29 | 0.07 | 0.51 |
| Salt | 4.5 | 1.8 | 4.6 | 1.7 | 4.5 | 1.8 | 0.05 | -0.16 | 0.27 | 0.06 | -0.18 | 0.30 | 0.11 | -0.14 | 0.36 | 0.21 | -0.03 | 0.46 |
| Sleep |  |  |  |  |  |  |  |  |  |  |  |  |  |  |  |  |  |  |
| Time spent asleep last night (hrs) | 6.6 | 1.3 | 6.5 | 1.4 | 6.5 | 1.3 | -0.15 | -0.33 | 0.02 | -0.09 | -0.27 | 0.08 | 0.03 | -0.16 | 0.23 | 0.09 | -0.11 | 0.28 |
| Average sleep duration (hrs/night) | 7.4 | 1.5 | 7.4 | 1.4 | 7.4 | 1.6 | -0.12 | -0.34 | 0.10 | -0.05 | -0.27 | 0.16 | -0.02 | -0.26 | 0.23 | -0.03 | -0.28 | 0.22 |
| Health-related quality of life | | |  |  |  |  |  |  |  |  |  |  |  |  |  |  |  |  |
| Summary mental component score (SF-8) | 50.4 | 9.6 | 51.2 | 8.8 | 50.6 | 9.2 | 0.49 | -0.72 | 1.70 | 0.56 | -0.72 | 1.83 | 0.43 | -0.97 | 1.83 | 0.54 | -0.89 | 1.97 |
| Summary physical component score (SF-8) | 48.3 | 9.5 | 48.7 | 9.0 | 48.5 | 9.4 | 1.07 | -0.11 | 2.25 | 0.57 | -0.65 | 1.80 | -0.31 | -1.61 | 0.99 | -0.59 | -1.94 | 0.76 |
| Summary index (EQ-5D-5L) | 0.8 | 0.2 | 0.8 | 0.2 | 0.8 | 0.2 | 0.02 | 0.00 | 0.04 | 0.00 | -0.02 | 0.02 | 0.00 | -0.03 | 0.03 | 0.01 | -0.02 | 0.03 |
| Self-related health (VAS) | 81.0 | 16.5 | 81.9 | 16.3 | 79.9 | 17.1 | 0.19 | -1.75 | 2.14 | 1.19 | -0.78 | 3.16 | -0.17 | -2.45 | 2.12 | -0.26 | -2.66 | 2.14 |
