# Supplementary material for: Promoting physical activity in a multi-ethnic population at high risk of diabetes: the 48-month PROPELS randomised controlled trial
Source: BMC Med. 2021 Jun 3;19:130. doi: 10.1186/s12916-021-01997-4 (PMC8173914; doi:10.1186/s12916-021-01997-4)
Supplement: Supplementary file 7 — Additional file 7:. Serious and non-serious adverse events. [file 12916_2021_1997_MOESM7_ESM.docx]

# **Additional file 7: Serious and non-serious adverse events**

|  |  | Treatment Group | | |
| --- | --- | --- | --- | --- |
|  | **Overall**  **(n=1366)** | **Control**  **(n=460)** | **Walking Away (n=450)** | **Walking Away Plus (n=456)** |
| Non-serious adverse events | 47 (3.44%) | 17 (3.70%) | 14 (3.11%) | 16 (3.51%) |
| Serious adverse events | 50 (3.66%) | 7 (1.52%) | 15 (3.33%) | 28 (6.14%) |

**Summary data**

#

# **Individual non-serious adverse events experienced by participants in the study, excluding the diagnosis of diabetes which is reported as a study outcome**

| Type of Adverse Event | Overall (n=1366) | Control  (n=460) | Walking Away (n=450) | Walking Away Plus  (n=456) |
| --- | --- | --- | --- | --- |
| Allergic reaction to penicillin | 1 (0.07%) | 1 (0.22%) |  |  |
| Allergic rhinitis | 1 (0.07%) | 1 (0.22%) |  |  |
| Arthritis | 1 (0.07%) |  |  | 1 (0.22%) |
| Broken bone | 3 (0.22%) |  | 2 (0.44%) | 1 (0.22%) |
| Cancer diagnosis | 2 (0.15%) |  | 1 (0.22%) | 1 (0.22%) |
| Carpal tunnel release surgery | 1 (0.07%) |  | 1 (0.22%) |  |
| Chest pain | 1 (0.07%) |  |  | 1 (0.22%) |
| Depression – suicidal thoughts | 1 (0.07%) |  |  | 1 (0.22%) |
| Exacerbated knee pain^a^ | 1 (0.07%) |  |  | 1 (0.22%) |
| Fall | 2 (0.15%) |  | 2 (0.44%) |  |
| Head injury (concussion) | 1 (0.07%) |  |  | 1 (0.22%) |
| Hip replacement (pre-planned) | 1 (0.07%) |  |  | 1 (0.22%) |
| M-skeletal injury to lower back and leg^b^ | 1 (0.07%) |  | 1 (0.22%) |  |
| Panic attack | 2 (0.15%) | 1 (0.22%) | 1 (0.22%) |  |
| Plantar fasciitis | 1 (0.07%) | 1 (0.22%) |  |  |
| Rash from activity monitor or dressing^c^ | 24 (1.76%) | 12(2.61%) | 5 (1.11%) | 7 (1.54%) |
| Tooth complaint | 1 (0.07%) | 1 (0.22%) |  |  |

Data presented as number of unique participants

^a^ AE related to the study intervention. Participant was withdrawn from the intervention.

^b^ AE related to the study intervention. Injuries occurred whilst increasing activity.

^c^AE related to study procedures, namely skin reaction to dressings used to fasten the activity monitor to leg or to the monitor itself
